# Predicting clinical trial success for *Clostridium difficile* infections based on preclinical data

**DOI:** 10.3389/frai.2024.1487335

**Published:** 2024-10-09

**Authors:** Fangzhou Li, Jason Youn, Christian Millsop, Ilias Tagkopoulos

**Affiliations:** ^1^Department of Computer Science, University of California, Davis, Davis, CA, United States; ^2^Genome Center, University of California, Davis, Davis, CA, United States; ^3^USDA/NSF AI Institute for Next Generation Food Systems, University of California, Davis, Davis, CA, United States

**Keywords:** machine learning, translational research, drug discovery, clinical trial, recommendation system

## Abstract

Preclinical models are ubiquitous and essential for drug discovery, yet our understanding of how well they translate to clinical outcomes is limited. In this study, we investigate the translational success of treatments for *Clostridium difficile* infection from animal models to human patients. Our analysis shows that only 36% of the preclinical and clinical experiment pairs result in translation success. Univariate analysis shows that the sustained response endpoint is correlated with translation failure (SRC = -0.20, *p*-value = 1.53 × 10^−54^), and explainability analysis of multi-variate random forest models shows that both sustained response endpoint and subject age are negative predictors of translation success. We have developed a recommendation system to help plan the right preclinical study given factors such as drug dosage, bacterial dosage, and preclinical/clinical endpoint. With an accuracy of 0.76 (F1 score of 0.71) and by using only 7 features (out of 68 total), the proposed system boosts translational efficiency by 25%. The method presented can extend to any disease and can serve as a preclinical to clinical translation decision support system to accelerate drug discovery and de-risk clinical outcomes.

## Introduction

*Clostridium difficile* is a spore-forming anaerobic bacteria widely distributed in the intestinal tract of humans and animals and in various environmental contexts (Smits et al., 2016). Over the past decade, the frequency and severity of *C. difficile* infection (CDI) have been increasing worldwide to become a leading nosocomial (hospital-acquired) pathogen (Czepiel et al., 2019). It is estimated to affect approximately 3 million individuals worldwide every year (Cole and Stahl, 2015), underscoring its significant public health impact. Although various treatments, such as metronidazole and oral vancomycin (Zar et al., 2007; Johnson et al., 2014; Teasley et al., 1983), have been approved for CDI management, the sustained efficacy, the effectiveness of treatment after the treatment is no longer administered, is low (Van Giau et al., 2019). This is particularly concerning given the recurrent nature of CDI (Cole and Stahl, 2015; McFarland et al., 1999), where the sustainability of treatment efficacy (the ability to prevent recurrence post-therapy) is crucial.

A predominant challenge in the development of treatments for *Clostridium difficile*, as with many diseases, lies in the limited rate of translational success from preclinical to clinical stages. For example, the chance of a potential drug candidate identified in the preclinical trials demonstrating efficacy in human studies and ultimately receiving approval is a mere 0.1% (Seyhan, 2019). Therefore, the development of a new drug is a time-consuming and costly process that often takes an average of 13 years and costs approximately US$1 billion (Ciociola et al., 2014) from the preclinical testing stage to FDA approval. The major causes for such translation failures are the lack of appropriate animal models for predicting the efficacy of the drug in humans (Seyhan, 2019; Paul et al., 2010), concerns for the efficacy and safety of the drugs (Kola and Landis, 2004), poor study design, ineffective site selection, poor recruitment, patient burden, and poor trial execution (Fogel, 2018). Efforts to enhance translational success have included the use of humanized animals, which exhibit more human-like responses to medical interventions (Shultz et al., 2007), and the application of biomarkers to reduce subjectivity in evaluating drug efficacy and safety (Yu, 2011). Machine learning-based approaches (Shah et al., 2019; Toh et al., 2019; Gayvert et al., 2016) have also been explored, predominantly focusing on attrition rates across different phases of clinical trials. However, these approaches often lack explainability due to the ‘black box’ nature of the models employed, interfering with their application in decision-making with high stakes (Lipton, 2017). Although machine learning models have shown promising results in other areas of life sciences (Lysenko et al., 2018; Wang et al., 2020; Eetemadi and Tagkopoulos, 2019), their application in bridging the gap between preclinical and clinical outcomes is hindered by a scarcity of expert-curated and harmonized datasets (Austin, 2021). This limitation is particularly pronounced in the context of *C. difficile*, where the complexity of the disease and its treatment modalities necessitates highly specialized and accurate data for effective model training and validation.

**Figure 1:**
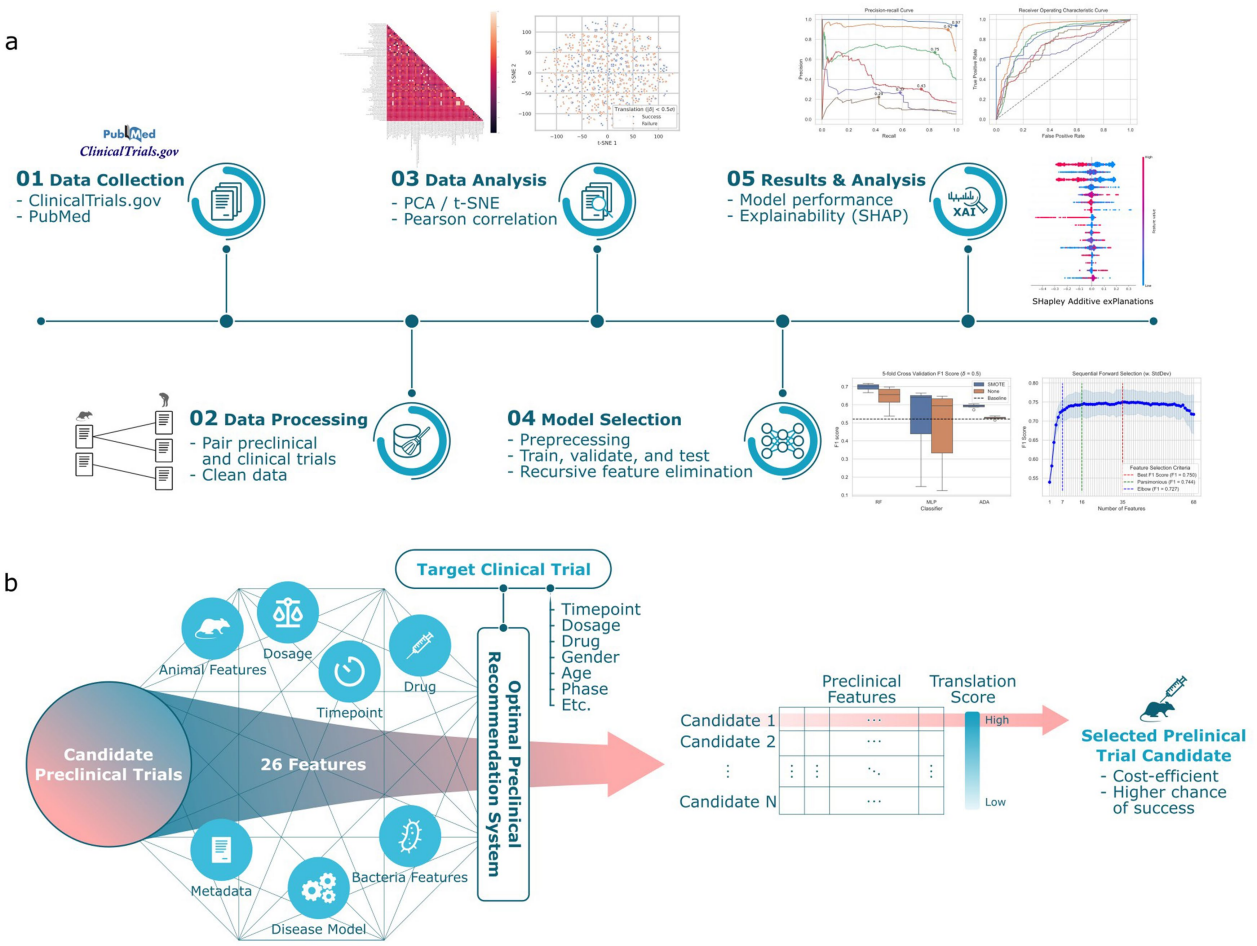
Overview of the preclinical recommendation system. (a) We collect data from publicly available preclinical and clinical trial information about *Clostridium difficile* infection. This dataset, designated as A2H, is constructed by pairing the preclinical trial with the clinical trial that shares the same drug. A binary classification label is applied to each pair, where a translation is successful (label 1) if the preclinical survival rates and clinical recovery rates are within a threshold δ . Then, a machine learning pipeline chooses the best combination of feature selection, missing value imputation, outlier detection, and classifier. We report the model performance and feature interpretation and predictions. (b) For any specified clinical trial of interest, our system computes a translation score for each candidate preclinical trial. This score quantitatively assesses the potential for successful translation. The preclinical trial that emerges with the highest translation score is then preferentially chosen to inform the design of the ensuing preclinical study.

In this study, to address the data scarcity, we manually curate the Animal-to-Human (A2H) translation dataset by extracting and pairing preclinical and clinical data for *C. difficile* infections from the scientific literature and ClinicalTrials.gov, respectively. Using our A2H dataset, we train a machine learning-based classifier to predict translational success (Figure 1a). Next, to address model interpretability, we apply an explainable AI method (Lundberg and Lee, 2017), and then we expand this predictor to a recommendation system (Figure 1b).

## Materials and methods

### Raw data acquisition

Clinical trial data about *C. difficile* infection (CDI) were collected from *ClinicalTrials.gov*, a comprehensive database of privately and publicly funded clinical studies. This study focused exclusively on completed interventional clinical trials that have published results to ensure the reliability and validity of the data. Parallel to clinical trial data collection, a thorough search was conducted on PubMed to identify publications that tested the same intervention (i.e., drug candidate) in an animal model as one of the clinical trials in our collection. Note that within the scope of a single trial, multiple experimental arms may be present, each contributing to the collective dataset. Here, an ‘arm’ is delineated as a cohort or subset of subjects receiving a particular therapeutic regimen (Ventz et al., 2018; Clinical and Designs, 2019). For instance, if a trial investigates two distinct treatment dosages, each dosage arm is a cohort that can have multiple individuals (or samples; animals for preclinical and humans for clinical studies, respectively). This resulted in a preclinical dataset of 480 arms from 43 preclinical trials, collectively consisting of 3 animal species, 60 interventions (drug candidates), and 29 variables. Similarly, the clinical dataset has 158 arms from 52 clinical trials, collectively consisting of 53 interventions (drug candidates) and 21 total variables. The raw data and variable description can be found in Supplementary Data 1.

### Data compendium

Due to the different interests in the endpoints measured in animal and human subjects, the number of preclinical and clinical trials that share the same endpoints is limited. For example, the survival rate is predominantly measured in preclinical trials, while the recovery rate is more often used in clinical trials for CDI. The survival rate indicates the ratio of living subjects, while the recovery rate indicates the ratio of healthy patients in the group at the point of measure. These two rates both reflect drug efficacy (Zhuang et al., 2009), making them more comparable and relevant for evaluating the effectiveness of treatments in both preclinical and clinical trials. In this section, we describe a strategy to derive translation outcome using these survival and recovery rates.

For preclinical studies, 480 arms with 27 variables were gathered, including specifics of animal (e.g., species, strain, sex, age, weight), disease model (e.g., administration sequence, disease strain), and drug (e.g., dosing, duration). For clinical studies, 272 arms with 15 variables were collected, encompassing aspects like dosage details, intervention class, therapeutic approach, and participant demographics. We then paired the preclinical and clinical trial arms that tested the same intervention (drug candidate) to construct an A2H dataset, which consists of 6,918 samples and 42 variables (27 from preclinical trials and 15 from clinical trials) after data cleaning and dropping 8 variables from the raw datasets (Supplementary Information Section 1.1; Supplementary Data 2). To analytically assign the binary dependent variable, we first calculated the difference between recovery and survival rates, denoted as δ (Equation 1), for each sample in the paired dataset as follows:


(1) $$-1.0\leq \delta =r_{r}-r_{s}\leq 1.0,$$

where $0.0\leq r_{s}\leq 1.0$ is the survival rate for animal subjects in the preclinical study, and $0.0\leq r_{r}\leq 1.0$ is the recovery rate for human subjects in the clinical study. We then fit a normal distribution (Equation 2) to these deltas as


(2) δ~N(μ,σ),

where mean ( μ ) and standard deviation ( σ ) estimate the standard distribution of δ . We assigned the binary label as follows:


(3) $$1\;\left(translation\;success\right):|\delta |<c*\sigma ,\;0\;\left(translation\;failure\right):|\delta |\geq c*\sigma ,$$

where c is a coefficient that controls the strictness of the translation success (Equation 3). We visualized our performance statistics using the A2H dataset with labels assigned with c=0.5 . However, different choices of c were also analyzed and reported (Supplementary Figure 1).

### Model selection

To find the most predictive machine learning model for our preclinical-to-clinical translation, we implemented a model selection pipeline that chooses the best data preprocessing combination and classifier. The categorical variables were transformed into input features applicable to machine learning using one-hot encoding. The pipeline includes, in the order specified, 2 feature scaling [Standard (Pedregosa et al., 2011) and MinMax (Pedregosa et al., 2011)], 1 missing value imputation (MVI) method [Simple (Pedregosa et al., 2011)], 1 oversampling (OS) [SMOTE (Chawla et al., 2002)], and 3 classifiers (CLS) [random forests (Breiman, 2001), AdaBoost (Freund and Schapire, 1997), MLP (Hinton, 1990)]. We rigorously tested each possible permutation of these preprocessing steps combined with a classifier using a 5-fold cross-validation approach to ensure robust evaluation, where each split was stratified, and samples from the same preclinical and clinical pair were grouped while splitting (Supplementary Information Section 1.2). Moreover, a grid search was performed on the classifiers to find the optimal hyperparameters using the validation set. Ultimately, the model candidate with the highest F1 score was selected as the best model. We have provided more in detailed information in Supplementary Information Section 1.3.

### Model interpretability

To increase the interpretability of the model, we applied the Shapley Additive Explanations (SHAP) (Lundberg and Lee, 2017) algorithm. The greater the magnitude of the SHAP value of a feature, the more influence that feature has on the model output. SHAP can provide the local explanation for each sample and the global explanation for an entire class by summarizing the overall importance of features across all data points. In this study, we used SHAP to analyze features that are influential in general to determine translation success.

## Results

### Experimental features correlated to preclinical to clinical translation success

**Figure 2:**
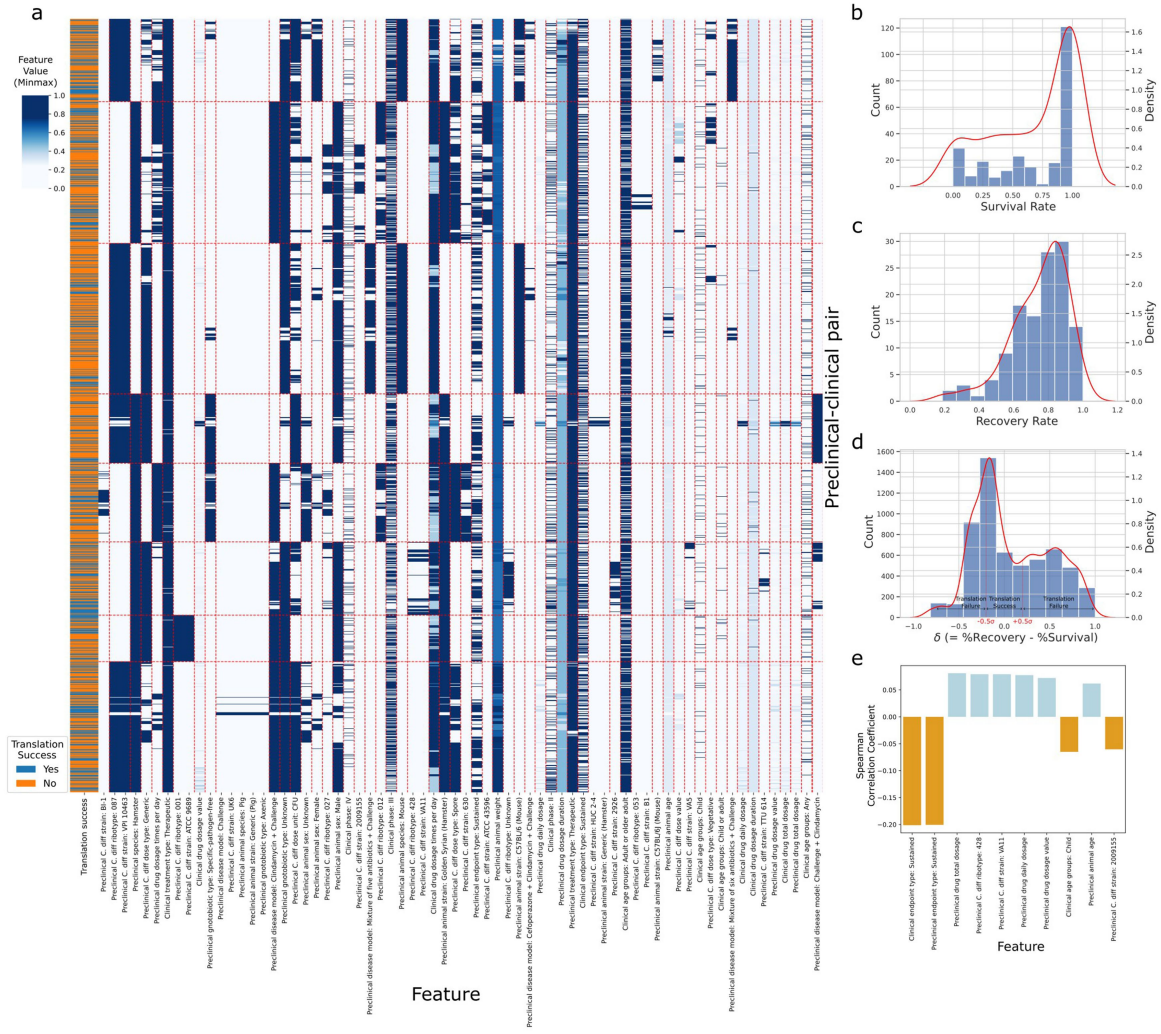
Statistics of the A2H dataset. (a) The spectral biclustering (*K_row_* = 8 and *K_col_* = 59) plot of the A2H dataset with |δ|<0.5σ . The vertical and horizontal red dashed lines separate column and row clusters, respectively. The features on the x-axis with colons in their names represent categorical features after one-hot encoding, and the string after the colon corresponds to the original category when the encoded feature is 1. The features without colons in their names represent numerical features, and the Min-Max scaling is performed on each numerical feature independently. (b) The distribution of the survival rate from the preclinical trial. (c) The distribution of the recovery rate from the clinical trial. (d) The distribution of delta $\delta =r_{r}-r_{s}$ , the difference between the clinical trial recovery rate and the preclinical trial survival rate. After fitting the normal distribution $\delta \sim N\left(\mu ,\sigma \right)$ to the delta, we label the preclinical/clinical trial pairs translation success (label 1) if δ lies between ±0.5σ around, and translation failure (label 0) otherwise. (e) Top 10 features with the highest absolute Spearman correlation coefficients for thresholds $\left|\delta \right|<0.5*\sigma$ , where δ is the different between clinical recovery and preclinical survival rates, and σ is the standard deviation of δ . All the features have adjusted *p*-value <0.001.

Figure 2a depicts the spectral biclustering (Kluger et al., 2003) of the 5,851 preclinical-clinical pair samples, excluding control intervention and inconvertible unit samples from the original 6,918, across the 68 features after performing one-hot encoding to the original 42 variables. From top to bottom, the fourth and sixth row clusters were associated with the lowest and highest average translation success rates of 0.25 and 0.45, respectively. We found that the row cluster with the lowest average translation success rate differentiated from other clusters due to its unique disease model, which challenged first and then treated animals with clindamycin (*p*-value<4.77 × 10^−122^). Similarly, the cluster with the highest average translation success rate had adopted *C. difficile* strains (e.g., VA11, 2,926, VA5, TTU 614) that were significantly different from those used in other clusters (*p*-value<7.2 × 10^−13^). A t-SNE plot for the A2H dataset can also be found in Supplementary Figure 2. The distribution of success metrics in both preclinical and clinical trials, specifically focusing on the survival and recovery rates, respectively, are shown in Figures 2b,c. These rates are skewed toward the right, partly due to the use of existing drugs like vancomycin and metronidazole as controls in case–control studies (Kaye et al., 2005). Delta ( δ ), the difference between the recovery rate and survival rate used to assign the target variable (translation success/failure) (see Methods), was modeled using a normal distribution as δ~N(0.09,0.41) (Figure 2d). We labeled the preclinical and clinical trial pairs (see Methods) that fell within ±0.5 standard deviation of δ=0.0 as ‘translation success.’ (3,746 samples) and ‘translation failure’ otherwise (2,105 samples) (Figure 2d). Spearman correlation coefficient of the features with the dependent variable lists 8 preclinical features as the top 10 most correlated features (Figure 2e), among which sustained response endpoint (i.e., outcome measured 14 days after the treatment) of the clinical trial was most negatively correlated to translation success (SRC = -0.20, *p*-value = 1.53 × 10^−54^).

### Machine learning models accurately predict translation success

**Figure 3:**
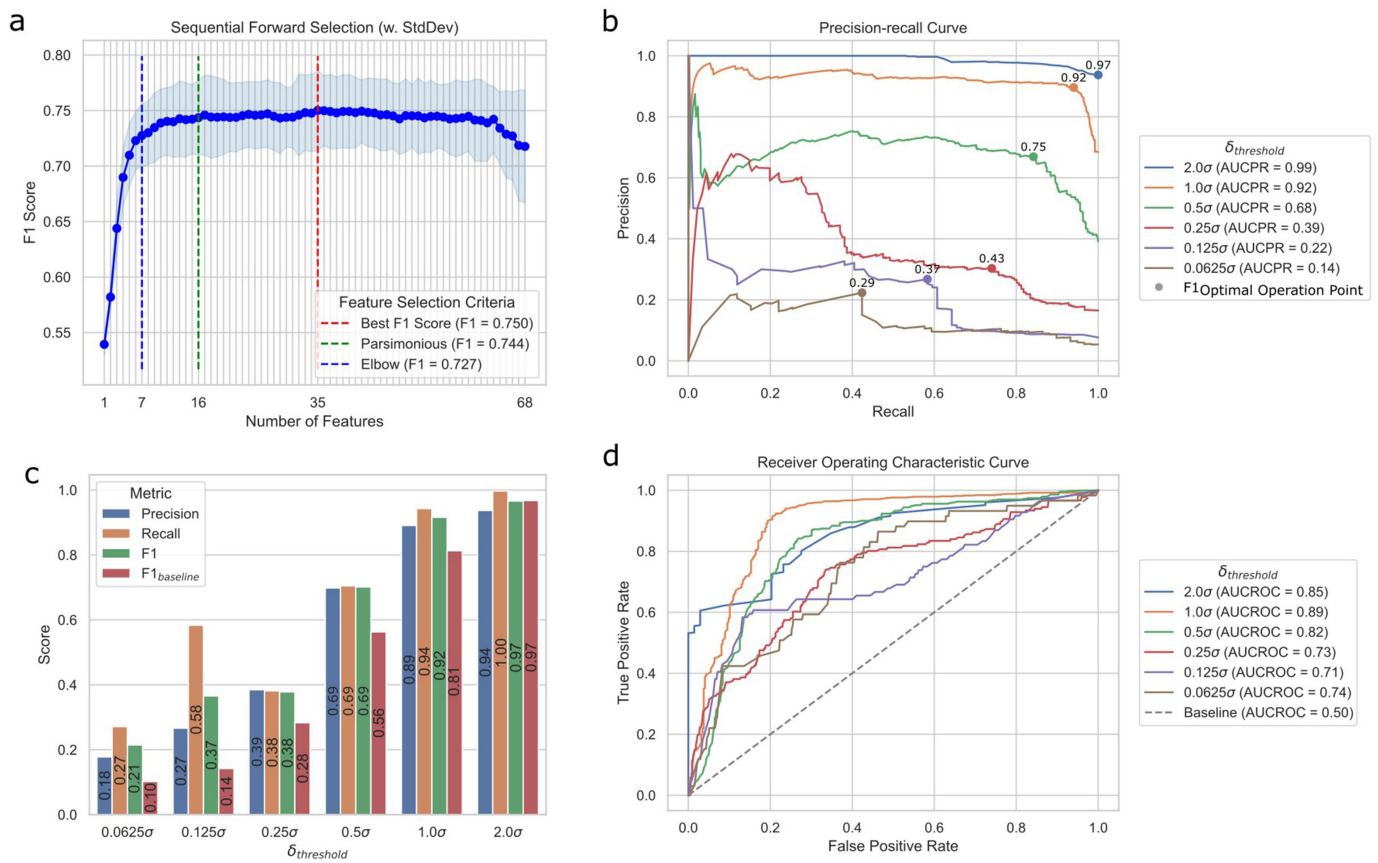
Prediction performance of the ML translation predictor. (a) Sequential feature selection results for different modes of selection criteria using the validation set. *Best* is the smallest feature subset when the F1 score was the best, *parsimonious* is the smallest feature subset that is within one standard error of the cross-validation performance, and *elbow* is the smallest feature subset based on the Kneedle method. (b) Precision-recall (PR) curves of different δ cutoff thresholds on the test set. F1_OOP_ denotes the optimal operating point chosen based on the best F1 score. The values above the dots indicate the F1 scores of OOPs. AUCPR stands for the area under the PR curve. (c) Performance metrics of different datasets created with different δ cutoff thresholds on the test set. (d) Receiver operating characteristic (ROC) curves of different δ cutoff thresholds based on the test set. AUCROC stands for the area under the ROC curve.

We implemented the model selection pipeline on A2H datasets created using different translation thresholds c (0.0625, 0.125, 0.25, 0.5, 1.0, and 2.0) (see Methods). In every scenario during the cross-validation process, the random forest model emerged as the top-performing classifier (Supplementary Table 1). Notably, we observed an improvement of the F1 score when applying SMOTE, especially for thresholds defined by smaller *c* (F1 improved 126.3, 124.9, 39.3, and 2.7% for *c* of 0.0625, 0.125, 0.25, 0.5, respective; Supplementary Figure 3). Running the sequential feature selection (Raschka, 2018) (SFS) in a parsimonious setting (smallest feature subset that is within one standard error of the best cross-validation F1 score) on the best pipeline with c=0.5 (FS: none, MVI: Simple, OS: SMOTE, CLS: random forest) significantly reduced the required number of features by 76.5% from 68 to 16 with negligible 0.8% performance loss (validation set F1 score decrease from 0.75 to 0.74) as shown in Figure 3a, where the results for other value of c can be found in Supplementary Figure 4. Moreover, we were able to achieve validation set F1 score of 0.73 with only 7 features identified using the Kneedle elbow method (Satopaa et al., 2011; Figure 3A). Table 1 further shows the holdout test set performance for different numbers of features for the best model. The best model pipeline for the benchmark A2H dataset ( c=0.5 ) on the holdout test set achieved a 25% better F1 score than a random baseline (0.69 vs. 0.56, respectively), while AUCPR and AUCROC were 0.68 and 0.82, respectively (Figures 3b–d). For all six different translation thresholds c except when c=2.0 , we had better performance than the random baseline (Figure 3c; Supplementary Table 2).

**Table 1:** The holdout test confusion matrix for the best translation model with |δ|<0.5σ .

Model	TP	FN	FP	TN	Precision	Recall	F1	Accuracy
Baseline	**430**	**0**	668	0	0.39	**1**	0.56	0.39
Random forest								
Best (*K* = 35)	298	132	**121**	**547**	**0.71**	0.69	0.70	**0.77**
Parsimonious (*K* = 16)	303	127	131	537	0.70	0.70	0.70	**0.77**
Elbow (*K* = 7)	335	95	173	495	0.66	0.78	**0.71**	0.76

The baseline model would always predict positive (translation success). For the random forest model, we showed three scenarios with different feature selection criteria. *K* denotes the number of features selected from the total 68 features. While deploying the model in real life, fewer features would reduce data collection costs. For each metric, the best performance was highlighted in bold, and the second best was highlighted with an underscore.

### Sustained response endpoint and subject age as predictors of translation success

**Figure 4:**
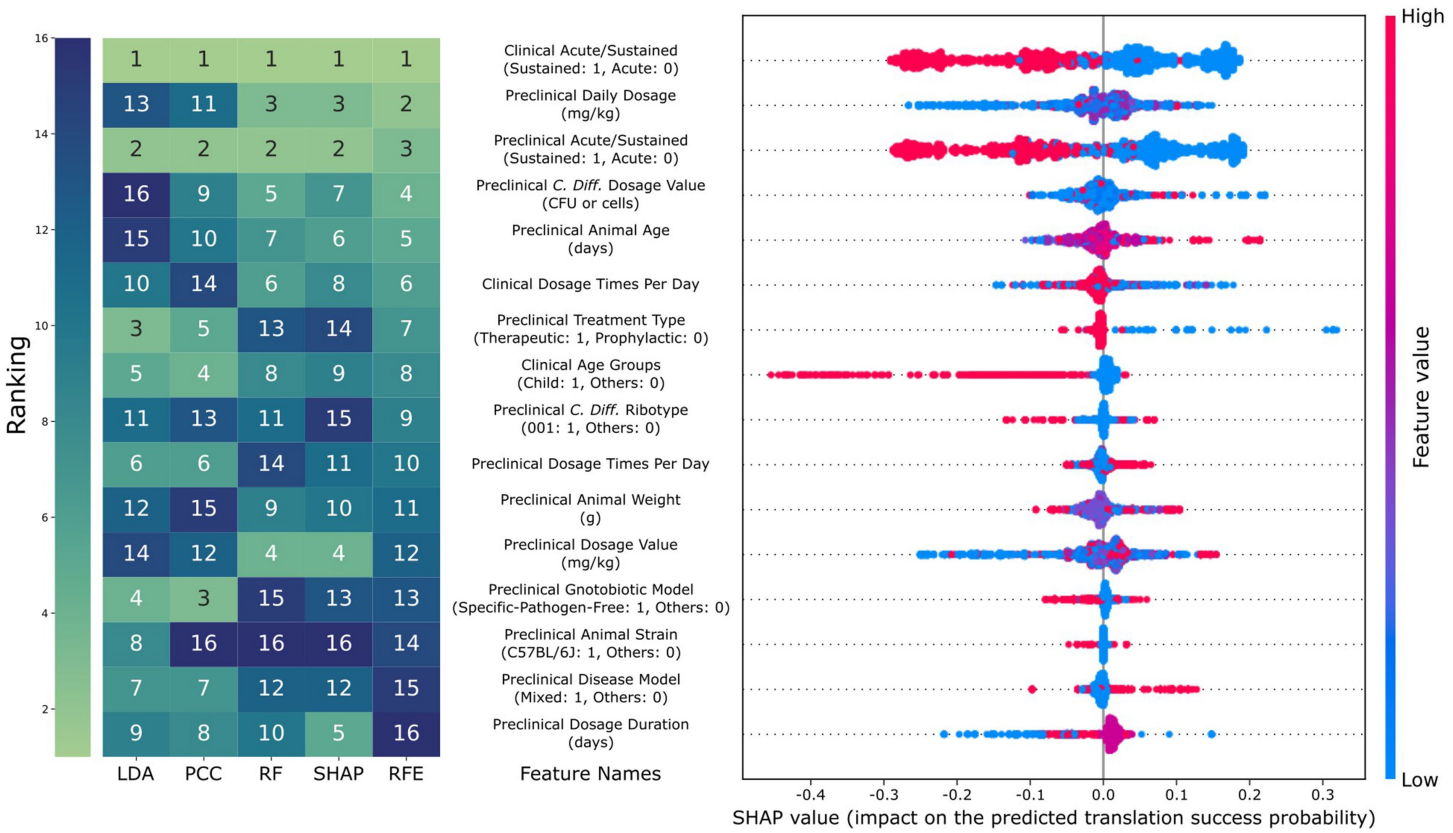
Comparison of feature importance and SHAP results. The rank of the 16 features, as selected using parsimonious selection criteria of the sequential feature selection, using different analysis methods (LDA: rank based on the absolute value of linear discriminant analysis, PCC: rank based on the absolute value of Pearson correlation coefficient, RF: rank based on random forest, and SHAP: rank based on the absolute value of SHAP). For the beeswarm plot on the right, each dot represents a data point, where red and blue correspond to high and low feature values, respectively. SHAP value indicates the amount of increase in translation success probability the feature causes for that data point. For example, a red dot with a positive SHAP value means that having a high feature value has a positive impact on predicting translation success for that data point.

We analyzed the feature importance of the best model for each c using five ranking methods: sequential feature selection, linear discriminant analysis (LDA), Pearson correlation coefficient (PCC), impurity-based feature importance of random forest (RF), and SHAP as shown in Figure 4a for c = 0.5. Of the 16 features selected by SFS, only three were from clinical features. The five ranking methods consensually identified whether clinical and preclinical endpoints were sustained or acute as most influential to the translation prediction (mean rank = 1 and 2.2). We found that RF and SHAP could highlight the importance of dosage-relevant features, while linear methods like LDA and PCC could not. A further investigation of SHAP values provided more detailed insights into the relationship between feature values and their impact on predictions. Specifically, the model considered sustained preclinical and clinical endpoints would decrease the translation success probability (mean SHAP value = −0.14 for both). This observation can be explained by the significantly lower translation success for samples with sustained preclinical and clinical endpoints compared to those with at least one acute endpoint (*p*-value = 3.3 × 10^−10^). The model also considered younger subjects for both animals and humans would be more likely to result in translation failure, with the animal age being highly correlated with the SHAP value (p-value = 3.9 × 10^−298^), with a smaller animal age value resulting in a more negative impact on translation success probability. Also, for the human subjects, the SHAP value of the child age group was significantly smaller than the more-aged group (mean SHAP value = 0.01 vs. -0.18; *p*-value = 8.7 × 10^−164^). The SHAP performance for other c can be found in Supplementary Figure 5.

## Discussion

Our research underscores the importance of refined preclinical strategies in drug development, a principle that holds true across various medical fields. The necessity for improved preclinical approaches, as indicated by the frequent phase III failures due to a lack of responder hypothesis-based trials (Sun et al., 2022), aligns with our findings, where a machine learning model driven by a selective feature set significantly enhanced the predictability of translational success.

Our choice to focus on *C. difficile* in this study stems from several key considerations. Firstly, the existence of well-established rodent models for *C. difficile* infection closely mimics the human disease and provides a robust basis for preclinical studies, therefore allowing for more accurate predictions of clinical outcomes. Additionally, the pressing need for improved treatment strategies for *C. difficile* infections, given their increasing prevalence and public health impact, underscores the practical significance of our research. Furthermore, the localized nature of *C. difficile* infections in the gut (Best et al., 2012), as opposed to systemic diseases, presents a unique opportunity. It allows for more controlled study parameters and a clearer understanding of treatment effects, which are critical for the successful application of machine learning techniques in predicting translational outcomes. This aspect is particularly vital in lightening the complexity that often accompanies the study of systemic diseases, where multiple organ systems and a myriad of physiological factors can confound results (Manor and Lipsitz, 2013).

There are a few areas of improvement. First, we assumed a direct and linear relationship between preclinical survival rates and clinical recovery rates. Yet, it is important to acknowledge that these metrics, while informative, may not fully capture the multifaceted nature of trial outcomes. Future studies could benefit from incorporating additional endpoints, such as percent weight change, patient-reported, and quality-of-life assessments, to provide a more comprehensive evaluation of trial success as well as the clinical utility. This, however, would result in a more complicated definition for translation success, which, in the future, would require us to provide better explanations for our recommendations such that they would be trustworthy and actionable for drug development researchers. Second, rather than employing the delta δ , which represents the difference between survival and recovery rates in current work, we could involve using a ratio of these rates. This change would be significant because a 10% difference in lower rates has different implications compared to a 10% difference in higher rates. Third, due to the complicated nature of (pre) clinical experimental designs, comparing results across different studies may have confounding biases resulting from unobserved variables. In this work, for example, we directly modeled the translation between preclinical and clinical trials, while in reality, trials usually consist of comparator groups to account for experimental biases. As a future improvement, we plan to adapt a recent work that used pairwise meta-learning to allow the model to learn across different experiments efficiently (Feng et al., 2024). Fourth, we would like to dive deeper into analyzing the impact of dosage regimens on model predictions. During our SHAP analysis, we found that high dosage amounts in preclinical trials had a positive impact on the model predictions on translation success rate, while they had a negative impact in clinical trials. While interesting, SHAP might produce misleading explanations for highly correlated features, e.g., daily dosage amount vs. dosage times per day (Aas et al., 2021). Dosage information is essential in drug development, and we aim to conduct a focused, rigorous analysis of dosage regimens for our next study phase. Fifth, the primary challenge of the data curation process is its dependency on expert-guided manual data curation. The current random forest model trained on a small dataset outperformed the state-of-the-art deep learning models, such as neural networks. The advantage of deep neural networks is their capability to generalize the representation to transfer to similar domain datasets (LeCun et al., 2015). Implementing an automated data extraction pipeline, leveraging transformer-based large language models (LLMs)(Lee et al., 2019; Vaswani et al., 2017; Liu et al., 2023), would significantly enhance the efficiency of extracting data from existing literature. This enhancement would be beneficial not only for *C. difficile* infection but also for a broader range of bacterial diseases, such as streptococcal infections, tuberculosis, and salmonellosis. By creating a dataset enriched with multi-omics information for these diverse diseases, we can develop a more generalizable ML-based predictor that demonstrates higher performance. We also consider including more clinical variables, such as patient demographics and health conditions, to further enhance the capability of our predictor. Additionally, this enriched dataset would facilitate intra-clinical predictions, such as forecasting the outcomes of clinical trial phase 2 based on phase 1 data.

## Conclusion

This study aims to help translate preclinical findings to clinical outcomes for *Clostridium difficile* infections, leveraging machine learning to enhance predictive accuracy and interpretability. Our model identifies key factors influencing translational success, streamlining drug development for CDI and potentially other diseases. This approach not only promises more effective treatments but also exemplifies the transformative impact of integrating computational methods in modern medicine, paving the way for advancements in personalized healthcare. The source code for our A2H recommendations system can be found at https://github.com/IBPA/A2H.

## Data Availability

The datasets presented in this study can be found in online repositories. The names of the repository/repositories and accession number(s) can be found in the article/Supplementary material.
