# Supplementary material for: Predicting clinical trial success for Clostridium difficile infections based on preclinical data
Source: Front Artif Intell. 2024 Oct 9;7:1487335. doi: 10.3389/frai.2024.1487335 (PMC11496251; doi:10.3389/frai.2024.1487335)
Supplement: Supplementary file 3 [file Data_Sheet_3.docx]

Predicting Clinical Trial Success for *Clostridium difficile* Infections Based on Preclinical Data

Fangzhou Li^1,2,3†^, Jason Youn^1,2,3†^, Christian Millsop^1,2^, and Ilias Tagkopoulos^1,2,3*^

^1^ Department of Computer Science, University of California, Davis, Davis, CA, USA

^2^ Genome Center, University of California, Davis, Davis, CA, USA

^3^ USDA/NSF AI Institute for Next Generation Food Systems (AIFS), University of California, Davis, Davis, CA, USA

^†^These authors contributed equally to this work.

^*^Correspondence: itagkopoulos@ucdavis.edu

**SUPPLEMENTARY INFORMATION**

**Table of Contents**

[1 Supplementary Text 3](#_Toc156081256)

[1.1 Data processing 3](#_Toc156081257)

[2 Supplementary Figures 4](#_Toc156081258)

[3 Supplementary Tables 9](#_Toc156081259)

[4 References 11](#_Toc156081260)

# Supplementary Text

## Data processing

The raw preclinical trial dataset consists of 480 arms (samples) and 29 variables. Before pairing, we dropped two features *sample group size* and *outcome coun*t as they were used to derive the *result* feature by calculating the *outcome count* / *sample group size*, which translates to the survival rate of the animal samples. Similarly, for the raw clinical trial dataset that consists of 158 arms (samples) and 21 variables, we dropped the following 6 features: *URL, Title, sample group size, outcome count, intervention:Specific,* and *intervention:Variation*. The *intervention:Specific* and *intervention:Variation* features are synonyms commonly used along with their *intervention* feature value and were used to generate duplicate rows before pairing so that it could be paired to appropriate preclinical trial samples. The *sample group size* and *outcome count* features were dropped as they were also used to derive the *result* feature, similar to what was done in the preclinical trail dataset.

## Data splitting

While we have made good efforts to collect a wide range of experimental variables, due to the complicated nature of (pre)clinical trials, it would be challenging to completely remove confoundingness in the dataset. To alleviate the potential data leakage during the data splitting, we made sure that the data points from the same (pre)clinical trials were uniquely contained by a data split. Specifically, before assigning categorical labels (see **Main Text Equation 3**), we applied equal-interval binning ($n=3$) to the numerical translation difference $\delta$ to apply stratified data splitting to ensure each split contained a similar target distribution. Note that we applied binning before assigning categorical labels to make sure the data splitting was the same across different strictness coefficients $c\in[0.0625,2.0]$ such that the results were comparable. We also made sure that data points with the same preclinical-clinical pair IDs were grouped together so that the same group would be assigned uniquely to one data split. This grouping was done to ensure the training data points that shared the same experimental settings were not leaked to validation and holdout test sets. We used the *StratifiedGroupKFold* class method from the scikit-learn(Pedregosa et al. 2018) Python library. Ultimately, we used 20% of the data points as the holdout test set and 5-fold cross-validation within the remaining data points for model selection.

## Model selection

In this section, we provide more details and rationales regarding the model selection pipeline.

### Missing value imputation

Since the missing value ratios in our dataset were insignificant, we used simple imputation(Pedregosa et al. 2018) for its least computational requirement. The simple imputation applied mean imputation for numerical variables and mode imputation for categorical variables.

### Oversampling

For oversampling, we used the Synthetic Minority Over-sampling Technique (SMOTE) algorithm(Chawla et al. 2002) from the imbalanced-learn(Lemaître, Nogueira, and Aridas 2017) Python library. This algorithm augments the dataset by synthesizing data points with minority labels by considering their nearest neighbors so that a more balanced dataset will train the classifier. In our pipeline, we considered two choices for the oversampling: With and without SMOTE. We found that SMOTE greatly benefitted the classifiers when the translation success window was stricter (i.e., smaller $c$), resulting in fewer positive samples.

### Classification

To have a wide range of model selection coverage, we considered three popular archetypes of classifiers for tabular data: Random forest(Breiman 2001) for ensemble learning, AdaBoost(Freund and Schapire 1997) for boosting, and multilayer perceptron(Hinton 1989) for neural network method. All these classifiers were able to handle non-linearity in the dataset and were versatile to fit a given data distribution.

### Hyperparameter tuning

For each model (defined by the combination of preprocessing steps and a classifier), we performed a 5-fold grid search to search for the best hyperparameter set. The 5-fold data splits (See **Supplementary Information Section 1.2**) were maintained across all models for comparable results. The best model was chosen based on the highest average 5-fold F1 score, which was further used to evaluate against the holdout test set. See **Supplementary Table 3** for the grid search space for each classifier.

# Supplementary Figures


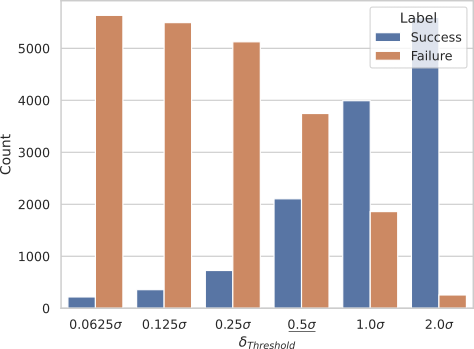


**Supplementary Figure 1.** Number of positives (translation success) and negatives (translation failure) of the whole dataset using different multipliers of standard deviation for thresholds. Note that we use $\pm0.5\sigma$ as the benchmark dataset.


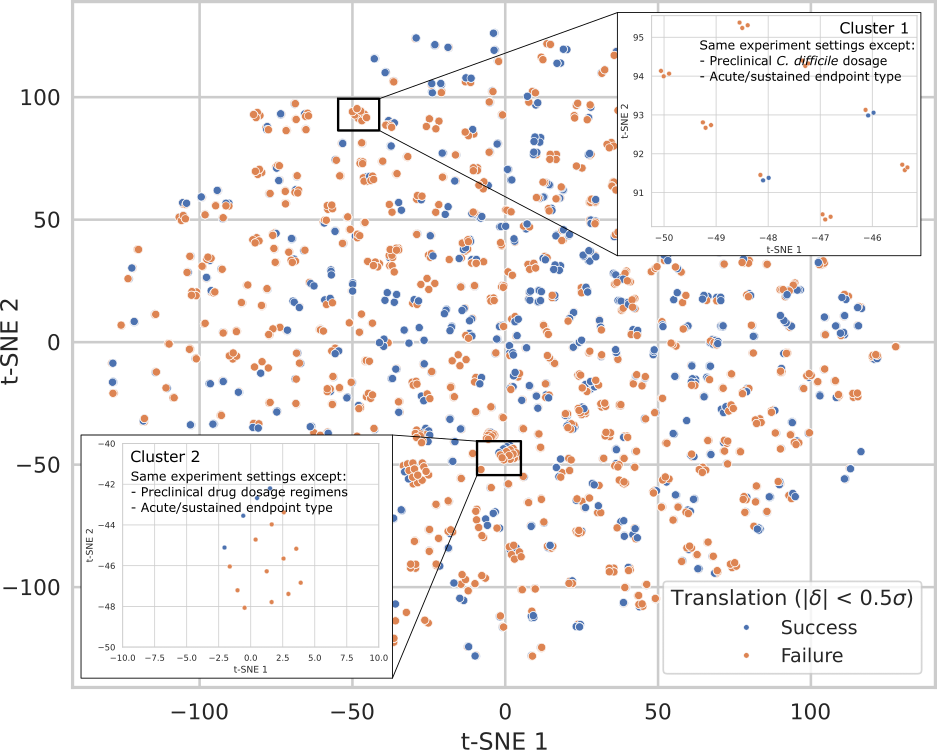


**Supplementary Figure 2.** The t-SNE plot of the A2H dataset with $|\delta|<0.5\sigma$. The first cluster shows a different *C. difficile* administration, and the second cluster shows different preclinical drug dosage regimens.


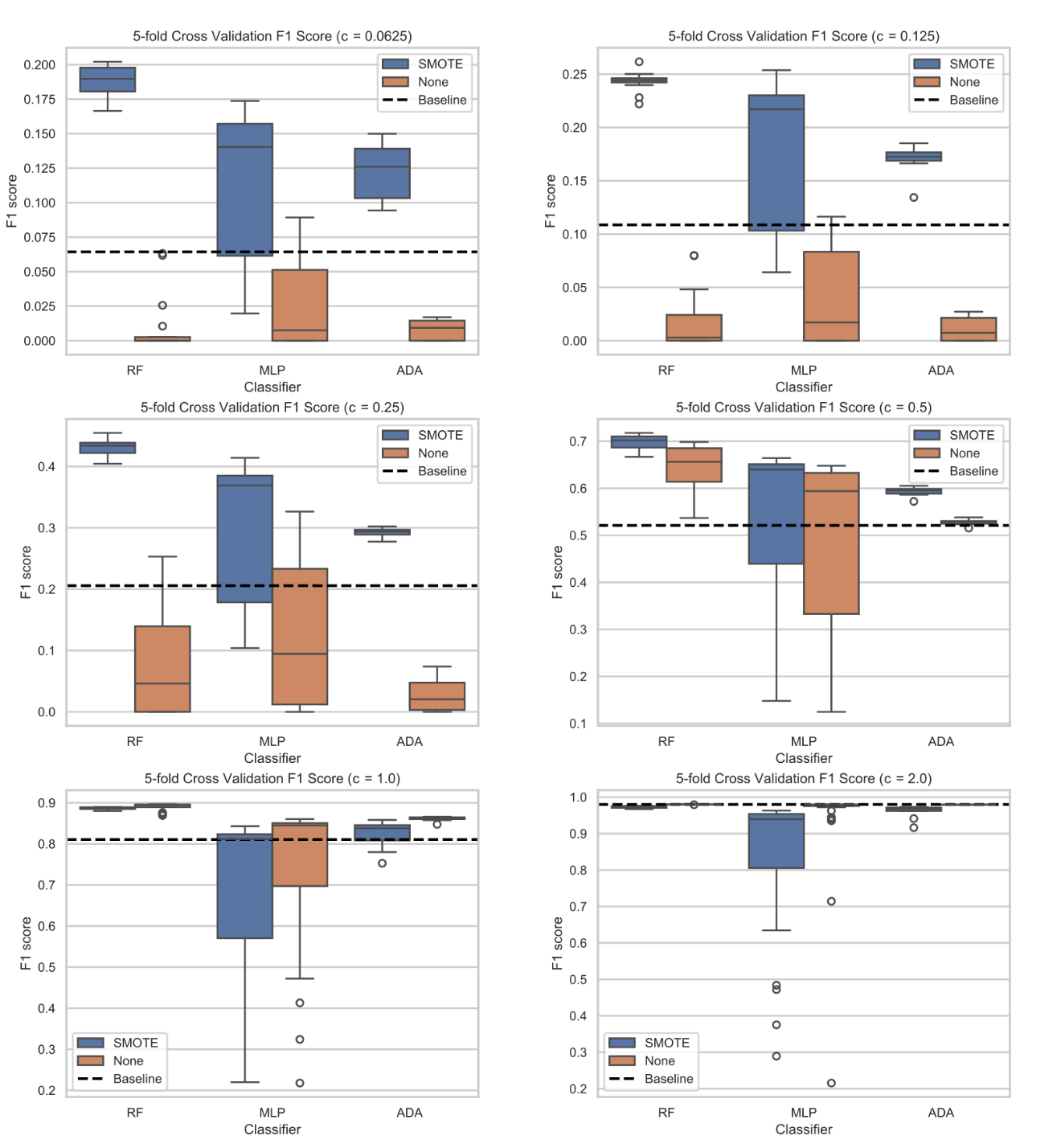


**Supplementary Figure 3.**

The model selection performance of each classifier for thresholds $\left| \delta\right|<c*\sigma$ with different $c$, where $\delta$ is the different between clinical recovery and preclinical survival rates, and $\sigma$ is the standard deviation of $\delta$. The baseline model is a dummy classifier, which always predicts positive.


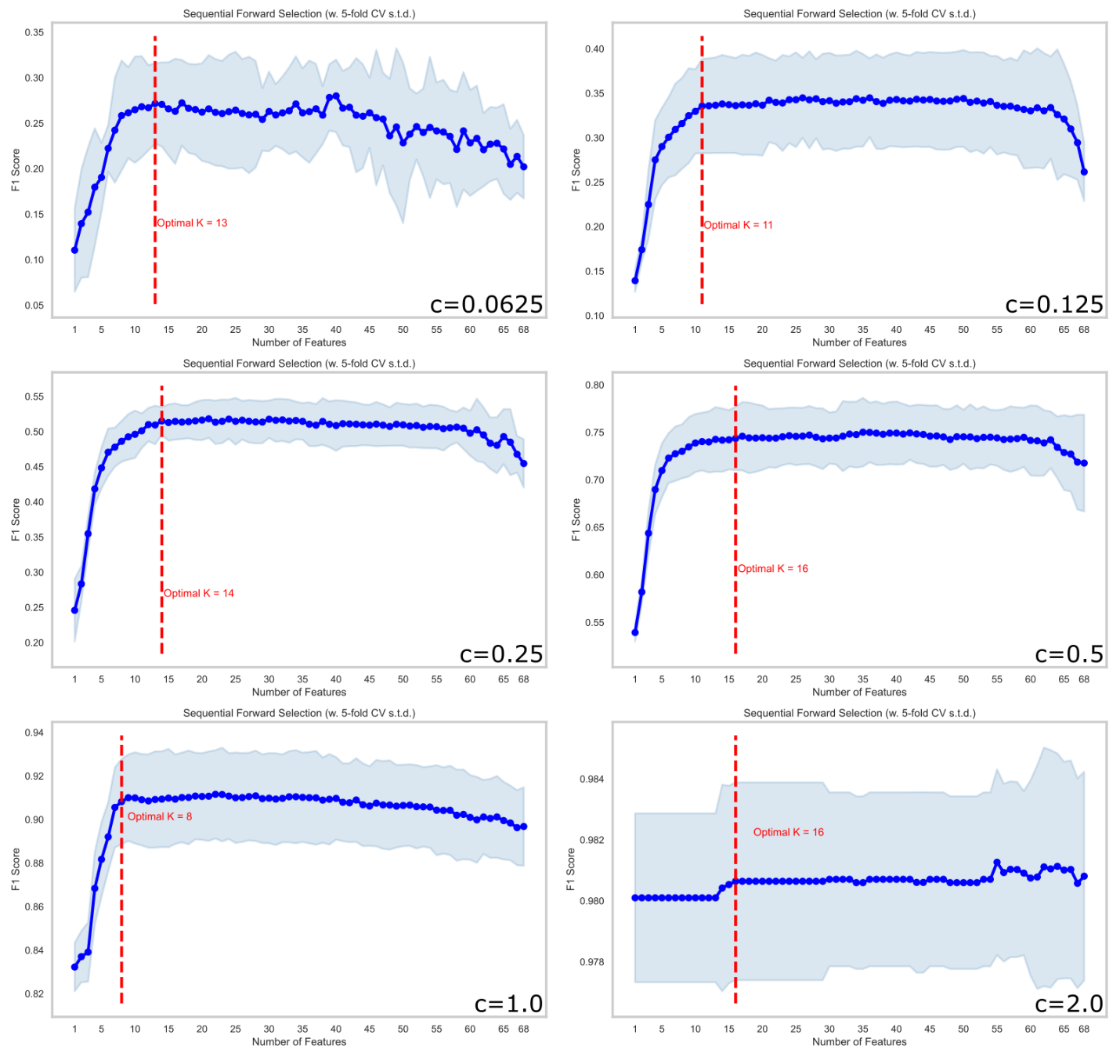


**Supplementary Figure 4.** The sequential feature selection performance of the best model for thresholds $\left| \delta\right|<c*\sigma$ with different $c$, where $\delta$ is the different between clinical recovery and preclinical survival rates, and $\sigma$ is the standard deviation of $\delta$. The optimal number of features K was found based on the parsimonious strategy defined in the Mlxtend library(Raschka 2018).


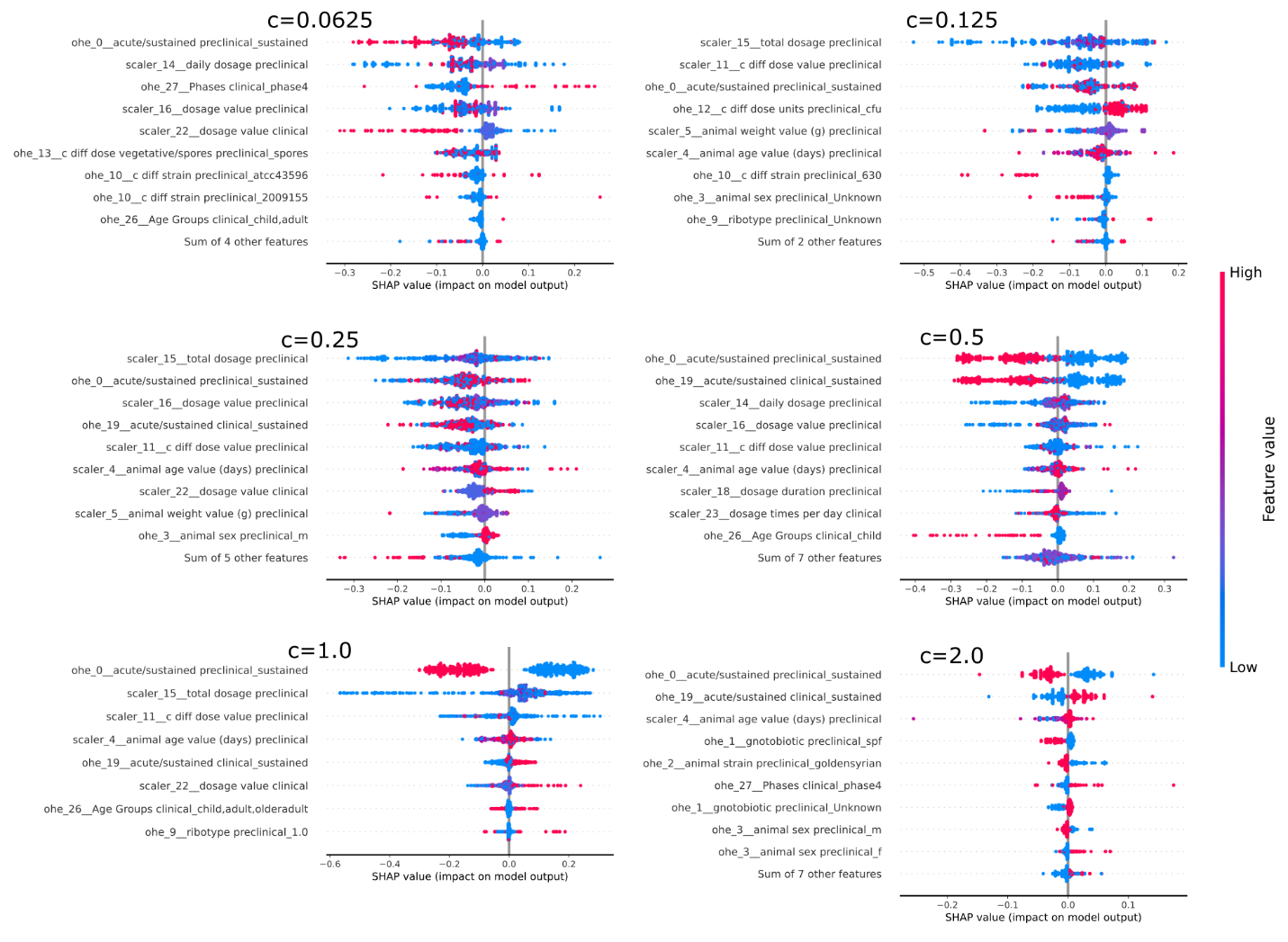
**Supplementary Figure 5.** The SHAP results of the best model for thresholds $\left| \delta\right|<c*\sigma$ with different $c$, where $\delta$ is the different between clinical recovery and preclinical survival rates, and $\sigma$ is the standard deviation of $\delta$. The feature uses the original name fed into the model, where the ‘ohe’ stands for *one-hot encoding*, and the last term after the underline indicates that the original feature value if the encoded feature is 1. Each point represents a sample, where the corresponding feature value is color-coded. The magnitude of the SHAP value of a sample indicates how impactful that feature was to the model’s prediction for that sample.

# Supplementary Tables

**Supplementary Table 1.** The 5-fold cross-validation performance of the best model for thresholds $\left| \delta\right|<c*\sigma$ with different $c$, where $\delta$ is the different between clinical recovery and preclinical survival rates, and $\sigma$ is the standard deviation of $\delta$. Feature scaling is not shown because it does not affect the random forest prediction.

| $\boldsymbol{c}$ | **Over-sampling** | **Classifier** | **Hyperparameters** | **Mean F1** |
| --- | --- | --- | --- | --- |
| 0.0625 | SMOTE | Random Forest | {'cls__criterion': 'entropy', 'cls__max_depth': 10,  'cls__max_features': 'log2', 'cls__n_estimators': 100} | 0.20 |
| 0.125 | SMOTE | Random Forest | {'cls__criterion': 'gini', 'cls__max_depth': 14,  'cls__max_features': 'log2', 'cls__n_estimators': 100} | 0.26 |
| 0.25 | SMOTE | Random Forest | {'cls__criterion': 'gini', 'cls__max_depth': 14,  'cls__max_features': 'log2', 'cls__n_estimators': 100} | 0.45 |
| 0.5 | SMOTE | Random Forest | {'cls__criterion': 'entropy', 'cls__max_depth': 14,  'cls__max_features': 'log2', 'cls__n_estimators': 100} | 0.72 |
| 1.0 | None | Random Forest | {'cls__criterion': 'entropy', 'cls__max_depth': 14,  'cls__max_features': 'log2', 'cls__n_estimators': 100} | 0.90 |
| 2.0 | None | Random Forest | {'cls__criterion': 'entropy', 'cls__max_depth': 14,  'cls__max_features': 'log2', 'cls__n_estimators': 100} | 0.98 |

**Supplementary Table 2.** The holdout testing confusion matrices of the best model after sequential feature selection for thresholds $\left| \delta\right|<c*\sigma$ with different $c$, where $\delta$ is the different between clinical recovery and preclinical survival rates, and $\sigma$ is the standard deviation of $\delta$.

| $\boldsymbol{c}$ | **TP** | **FN** | **FP** | **TN** | **Accuracy** | **Precision** | **Recall** | **F1** |
| --- | --- | --- | --- | --- | --- | --- | --- | --- |
| 0.0625 | 16 | 43 | 74 | 965 | 0.89 | 0.18 | 0.27 | 0.21 |
| 0.125 | 49 | 35 | 135 | 879 | 0.85 | 0.27 | 0.58 | 0.37 |
| 0.25 | 68 | 113 | 106 | 811 | 0.80 | 0.39 | 0.38 | 0.38 |
| 0.5 | 298 | 132 | 131 | 537 | 0.76 | 0.69 | 0.69 | 0.69 |
| 1.0 | 709 | 43 | 87 | 259 | 0.88 | 0.89 | 0.94 | 0.91 |
| 2.0 | 1026 | 3 | 69 | 0 | 0.93 | 0.94 | 1.00 | 0.97 |

**Supplementary Table 3.** Grid search space for hyperparameter tuning for each classifier. The grid search was implemented using the scikit-learn Python library. The parameters not mentioned for the classifiers implied the usage of the default values.

| **Classifier** | **Parameter Name** | **Parameter Space** |
| --- | --- | --- |
| Random Forest | # Estimators | 100, 200 |
|  | Criterion | ‘gini’, ‘entropy’ |
|  | Max Depth | 8, 10, 12, 14 |
| AdaBoost | # Estimators | 100, 200, 400 |
|  | Learning Rate | 0.1, 0.5, 1.0 |
|  | Algorithm | ‘SAMME.R’ |
| Multilayer Perceptron | Hidden Layer Sizes | (100,), (100, 100), (100, 100, 100) |
|  | Learning Rate | ‘adaptive’ |
|  | Learning Rate Init | 1e-5, 1e-4, 1e-3 |
|  | Max Iter | 1000 |
|  | # Iter No Change | 5 |

# References

Breiman, Leo. 2001. “Random Forests.” *Machine Learning* 45 (1): 5–32. https://doi.org/10.1023/A:1010933404324.

Chawla, Nitesh V., Kevin W. Bowyer, Lawrence O. Hall, and W. Philip Kegelmeyer. 2002. “SMOTE: Synthetic Minority over-Sampling Technique.” *J. Artif. Int. Res.* 16 (1): 321–57.

Freund, Yoav, and Robert E Schapire. 1997. “A Decision-Theoretic Generalization of On-Line Learning and an Application to Boosting.” *Journal of Computer and System Sciences* 55 (1): 119–39. https://doi.org/10.1006/jcss.1997.1504.

Hinton, Geoffrey E. 1989. “Connectionist Learning Procedures.” *Artificial Intelligence* 40 (1): 185–234. https://doi.org/10.1016/0004-3702(89)90049-0.

Lemaître, Guillaume, Fernando Nogueira, and Christos K. Aridas. 2017. “Imbalanced-Learn: A Python Toolbox to Tackle the Curse of Imbalanced Datasets in Machine Learning.” *Journal of Machine Learning Research* 18 (17): 1–5.

Pedregosa, Fabian, Gaël Varoquaux, Alexandre Gramfort, Vincent Michel, Bertrand Thirion, Olivier Grisel, Mathieu Blondel, et al. 2018. “Scikit-Learn: Machine Learning in Python.” arXiv. https://doi.org/10.48550/arXiv.1201.0490.

Raschka, Sebastian. 2018. “MLxtend: Providing Machine Learning and Data Science Utilities and Extensions to Python’s Scientific Computing Stack.” *Journal of Open Source Software* 3 (24): 638. https://doi.org/10.21105/joss.00638.
